# Unsegmented flow approach for on-line monitoring of pH, conductivity, dissolved oxygen and determination of nitrite and ammonia in aquaculture

**DOI:** 10.1155/S1463924694000052

**Published:** 1994

**Authors:** A. C. Ariza, P. Linares, M. D. Luque de Castro, M. Valcárcel

**Affiliations:** Department of Analytical Chemistry Faculty of Sciences University of Códoba Córdoba E-14004 Spain

## Abstract

A fully automated flow system for on-line monitoring of analytes/parameters of interest in aquaculture is described. The approach has been optimized for the photometric determination of nitrite and ammonia and the continuous monitoring of pH, conductivity and dissolved oxygen, but these analytes/parameters are readily changeable as required. The system has been tested by monitoring these species in the input and output sea water streams of tanks at a fish breeding farm and also by monitoring water containing high concentrations of fish feed.


Journal of Automatic Chemistry, Vol. 16, No. 2 (March–April 1994), pp. 59–62

### Unsegmented flow approach for on-line monitoring of pH, conductivity, dissolved oxygen and determination of nitrite and ammonia in aquaculture

**A. C. Ariza, P. Linares, M. D. Luque de Castro and M. Valcárcel**

*Department of Analytical Chemistry, Faculty of Sciences, University of Córdoba, E-14004 Córdoba, Spain*

*A fully automated flow system for on-line monitoring of analytes/parameters of interest in aquaculture is described. The approach has been optimized for the photometric determination of nitrite and ammonia and the continuous monitoring of pH, conductivity and dissolved oxygen, but these analytes/parameters are readily changeable as required. The system has been tested by monitoring these species in the input and output sea water streams of tanks at a fish breeding farm and also by monitoring water containing high concentrations of fish feed.*

### Introduction

Among the main targets of today's analytical chemistry are on-line monitoring and multideterminations because the demand for this type of measurement in the industrial and biotechnological fields is growing very rapidly. Continuous unsegmented flow techniques are clearly very useful in on-line monitoring and multideterminations, and a large number of applications have been reported in the last few years [1]. Most of these applications relied on either of the two major unsegmented-flow approaches: completely continuous flow analysis [2] and flow injection analysis [3,4]. These techniques can also be very valuable in the aquaculture field as they can provide excellent methods for species of interest which require continuous or periodical monitoring.

The aim of the research reported in this paper was to show the potential of continuous unsegmented techniques to develop methods of interest in aquaculture, which can be easily implemented in fish hatcheries. The methods proposed are based on the following principles:

(1) The on-line monitoring of sample properties such as conductivity, and direct measurements of pH and dissolved oxygen by placing the flow-cells/sensors suitable for each parameter in series in an output stream, which can be optionally returned to the overall system after monitoring.

(2) The on-line determination of nitrogen compounds, nitrite and ammonia, based on conventional photometric reactions and on the use of a specialized unsegmented flow single-channel manifold, the key component of which is a programmable switching valve which allows sequential selection of sample and the reagents required to implement given determinations. This approach was previously used [5] to develop methods for the determination of phosphate and nitrate in natural waters for subsequent application in aquaculture. This stream would not be returned to the system.

### Experimental

### *Reagents*

The stock solutions used included the Nessler reagent for ammonia (4% (w/v) mercury(II) chloride solution was added to 4 g of potassium iodide in 40 ml of distilled water, stirring gently until the appearance of a red precipitate). Then 100 ml of 2 N sodium hydroxide solution was added and the solution was diluted to 500 ml with distilled water; finally, a small amount of mercury(II) chloride solution was added until permanent turbidity was observed. The solution was allowed to stand overnight, then decanted and stored in topaz bottles. This solution was mixed in-line in a 1:1 ratio with another, prepared by suspending 0·5 g of poly(vinyl) alcohol in about 50 ml of distilled water under continuous stirring, then diluting to 100 ml with distilled boiling water. For nitrite, 0·380 g of sulphanilamide was dissolved in 4 ml of concentrated HCl and diluted to 100 ml with distilled water; and 0·140 g of N-/1-naphthylethylenediamine and 1·600 g of NaCl were dissolved in 100 ml with distilled water. Aqueous standard solutions of nitrite and ammonium ion were prepared from sodium nitrite and ammonium sulphate (supplied by Merck).

All chemicals used were analytical reagent grade.

### *Instruments and apparatus*

A Unicam 8625 UV-Vis spectrometer equipped with a Hellma 178·12QS flow-cell (inner volume 18 μl) was used. This was connected to a Radiometer pHM 62 pH-meter; a 4-channel Eppendorf Eva Peristaltic pump; and a 9-way 2500 Eppendorf Eva switching valve were used. A FIAtron 721 flow cell with a glass-calomel microelectrode connected to a Radiometer pHM 62 pH meter was used for pH determination, and a laboratory-made PTFE dissolved oxygen flow cell was connected to an YSI 5700 dissolved oxygen-meter equipped with an YSI 5739 sensor to measure oxygen. A laboratory-made conductivity flow cell, with platinum electrodes separated at an adjustable distance between 0·5 and 5·0 mm connected to an YSI 35 conductance meter, was used for conductivity measurement.

0142-0453/94 $10.00 © 1994 Taylor & Francis Ltd.

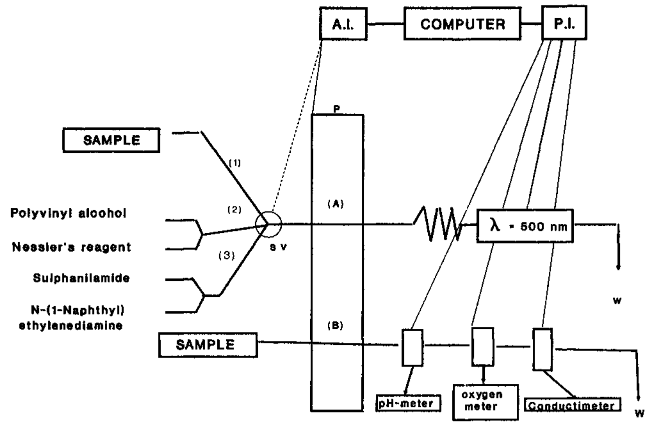

*Figure 1. General scheme for on-line monitoring of pH, dissolved oxygen, conductivity, nitrite and ammonia. S.V. denotes switching valve, P peristaltic pump, W waste, A.I. and P.I. active and passive interfaces, respectively.*

System control and data processing were done with a PC 80286, equipped with a PC-ADDA/14 analogue-to-digital interface with 12 bit resolution for the data acquisition, a dual 'serial-control' interface for the pump and valve multi input–output, a 40 MB hard disk and a $3\frac{1}{2}$ in floppy disk drive, and a STAR LC10. Tecator TMII chemifolds were used as confluence points.

### *Manifold*

Figure 1 shows the arrangement used in which the sample stream feeds channels A and B. The upper subsystem is devoted to nitrite and ammonia determination, the central element of which is the programmable switching valve. The insertion into the main channel (A) of the sample-reagents sequence gives place to the derivatization step prior to detection of the analytes. The sequence of the segments of fluid circulating along the main channel A depends on the position of the switching valve (SV), between 1 and 3, in order to establish the previously programmed reagent-sample sequence. By sequentially selecting channel 1 (sample) and 2 (Nessler reagent/polyvinyl alcohol solution) ammonia can be determined. The sequential change of reagents (sulphanilamine/N-(1-naphthyl)ethylenediamine) channel 3) enables the determination of nitrite. The PC acquires the absorbance signals through the passive interface and converts them into ammonia and nitrite concentrations. Sample running through channel B passes through the flow cell of the pH meter, oxygen meter and conductimeter, the data from each detector being collected by the computer and the value calculated after comparing the signal with the calibration curve. Streams along channels A and B run by aspiration through the peristaltic pump (P).

### *Software*

A BASIC program was developed to control the manifold and perform data acquisition and processing. The program offers the following features: selection of the intervals for switching of the selecting valve to each position; speed of the peristaltic pump and intervals for data acquisition from each detector; number of analyses to be carried out; interval between consecutive analyses; and display of the maximum absorbance of the peak recorded on each sample insertion in channel A. Samples were inserted in triplicate and the average absorbance was calculated by comparison with the stored calibration graph, the anlyte concentration was then displayed on screen. The data from the detectors in channel B were collected at preset intervals, depending on the change of the monitored parameter.

### Results and discussion

### *Sequential method for determination of nitrite and ammonia*

The determination of nitrite was based on the Griess diazotization reaction, with maximum absorption of the reaction product at 540 nm. The Nessler reaction for ammonia is normally monitored at 390 nm.

A compromise configuration was adopted for the sequential determination of both analytes. The features of the chemical and flow systems and the values of the optimized variables are listed in table 1. All variables were optimized by the univariate method by changing them within the intervals shown in table 1. The criterion for optimization was that the maximal height of the signal was obtained with minimal blank contribution. After optimization of each method, a compromise was adopted for common variables (such as temperature and wavelength) for monitoring. As the analytical signals for both determinations were only slightly influenced by changes in temperature in the range 10–50°C, analyses were performed at room temperature. 500 nm was the wavelength selected—at this value the loss of sensitivity of each method relating to its optimal wavelength was minimal.

*Table 1. Optimization of chemical and flow variables for the determination of nitrite and ammonia.*

| Chemical variables | | |
|---|---|---|
| Variable | Range studied | Optimum value |
| **Nitrite** | | |
| Sulphanilamide conc. | 2·0–8·0 g $l^{-1}$ | 4·0 g $l^{-1}$ |
| HCl conc. | 4·0–10·0% | 4·0% |
| N-(1-naphthyl)ethylenediamine | 2·0–8·0 g $l^{-1}$ | 6·0 g $l^{-1}$ |
| NaCl conc. | 1·5–3·5% | 2·5% |
| **Ammonia** | | |
| Nessler reagent | CONC.–1:10 | CONC. |
| NaCl conc. | 0·5–4.0% | 2.5% |
| **Flow variables** | | |
| Variable | Range studied | Optimum value |
| Flow rate | 0·5–2·0 ml $min^{-1}$ | 1·0 ml $min^{-1}$ |
| Time 2 | 4–15 s | 7 s |
| Time 3 | 15 s | 7 s |

Automatic control to the switching valve allowed the intervals for each position to be programmed, so the volume of sample and reagent could be changed as required. This allowed the sensitivity of the method to be changed from 0·200 AU (absorbance units) to 0·450 AU for a concentration of 1·5 μ $ml^{-1}$ of nitrite, and from 0·250 to 0·880 AU for 5·0 μg $ml^{-1}$ of ammonia when the sample/reagent volumes were changed from 83/1500 to 200/1500 μl. The ratio of concentrations in which ammonia and nitrite can be determined can also be changed in this way: the ratios can be changed from 1:25 to 6:1 nitrite:ammonia and adjusted to their normal concentration ranges in fish farms.

The transient signals provided by the photometer were independent of salinity, allowing analytes in sea water to be determined.

### Features of the determination

The features of the sequential determination of analytes are summarized in table 2. The sensitivity was acceptable in both cases and the determination ranges encompass the concentration required for monitoring fish hatcheries and tanks systems. The precision was also acceptable for these determinations. The analytes can be determined in triplicated insertion of each sample, at a rate of 40 samples per hour.

### On-line monitoring of pH, conductivity and dissolved oxygen

As shown in figure 1, these parameters can be monitored in a single line using continuous serial detectors. Calibration was as follows:

(1) *pH meter.* Two commercial standard buffers of pH 7·0 and 4·0 were used for calibration, by circulating each of these solutions through channel B at the flow rate at which the sample would be circulated (1 ml $min^{-1}$). The pH meter was adjusted to the pH of each buffer.

(2) *Oxygen meter.* Calibration was performed by comparing the values of oxygen concentrations obtained by a static method with those provided by the continuous system at the flow rate in channel B (1 ml $min^{-1}$). The correlation equation was:

$$Y = 6{\cdot}696 + 0{\cdot}575X \qquad (r^2 = 0{\cdot}9966)$$

where $Y$ is the value obtained by the static method and $X$ corresponds to the flow method.

(3) *Conductimter.* Standard solutions of NaCl, at concentrations between $1 \times 10^{-3}$ and $5 \times 10^{-1}$ M, were prepared, covering the ranges of natural and saline waters. The data obtained were consistent with those provided by the static method and also with those from the *Handbook of Chemistry and Physics* [6].

### On-line monitoring of real samples

The system was checked with two real samples.

(1) Sea water samples from a feed stream for nursery tanks. Inputs and outputs were continuously monitored during 30 min intervals over a two-week period. Results were compared with those of static measurements of pH, conductivity and dissolved oxygen, and with the daily batch analyses using conventional methods for ammonia and nitrite. The results were mutually consistent; they were also consistent with those of the batch and static methods, thereby showing the accuracy of the proposed methods and the stability of these analytes in the samples.

(2) Fish feed, consisting of fish flour and insect larvae of different textures and grain size, were suspended in tanks containing tap water (10 g of feed $l^{-1}$). The evolution of the tanks content was continuously monitored during 30 min intervals over a two-week period. Table 3 shows some of the values obtained (average of the measurements performed during the first 6 h of each day) for pH, conductivity and dissolved oxygen, and figure 2 shows the evolution of the evolution of the

*Table 2. Features of the sequential determination of nitrite and ammonia.*

| | Equation | Linear range μg $ml^{-1}$ | $r^2$ | RSD (%) |
|---|---|---|---|---|
| Nitrite | $h = 0{\cdot}070 + 0{\cdot}323\lvert NO_3^-\rvert$ | 0·5–3·0 | 0·9934 | ±3·0 |
| Ammonium | $h = 0{\cdot}216 + 0{\cdot}027\lvert NH_4^+\rvert$ | 0·5–7·0 | 0·9925 | ±2·5 |

*Table 3. Evolution of pH, conductivity and dissolved oxygen in the decomposition of fish feeds.*

| | pH | | | Conductivity | | | Dissolved oxygen | | |
|---|---|---|---|---|---|---|---|---|---|
| Day | 1st | 7th | 14th | 1st | 7th | 14th | 1st | 7th | 14th |
| 1 | 5·58 | 5·90 | 6·42 | 24·00 | 14·25 | 69·75 | 31·40 | 45·92 | 56·12 |
| 2 | 5·54 | 6·39 | 6·14 | 30·50 | 6·00 | 60·60 | 21·57 | 60·00 | 52·98 |
| 3 | 5·57 | 6·54 | 6·70 | 23·23 | 6·00 | 54·25 | 63·40 | 57·55 | 55·32 |
| 4 | 5·60 | 5·86 | 6·22 | 18·00 | 4·50 | 45·50 | 12·02 | 57·82 | 56·30 |
| 5 | 5·64 | 6·26 | 5·07 | 11·00 | 4·50 | 39·40 | 26·57 | 57·82 | 57·12 |
| 6 | 5·59 | 5·89 | 6·17 | 16·50 | 31·75 | 46·00 | 17·7 | 15·20 | 30·94 |

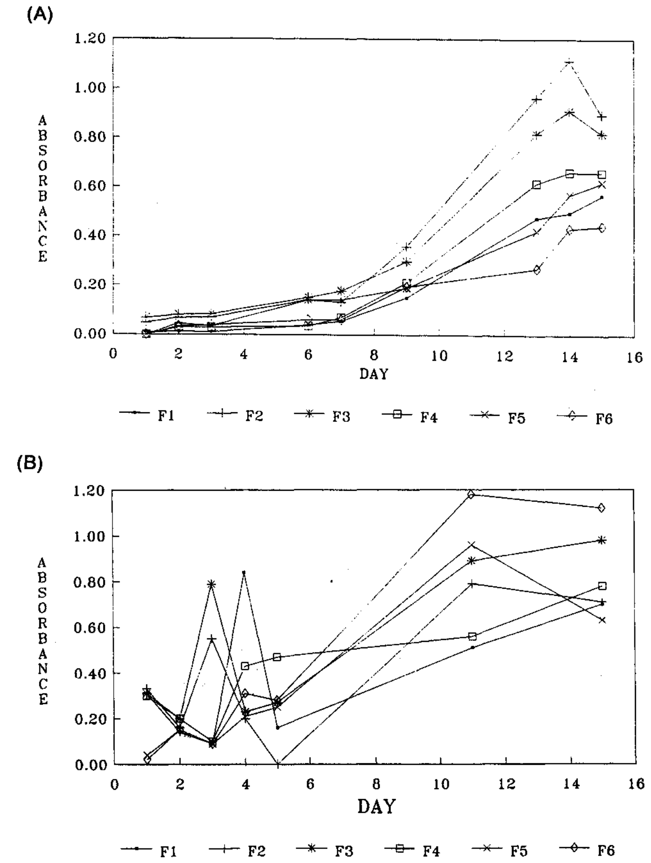

*Figure 2. Evolution of the ammonia (A) and nitrite (B) content from fish feed suspended in tap water over a two-week period.*

concentration of ammonia and nitrite during the monitoring period. Concentrations of ammonia and nitrite increased over the monitoring period as a result of feed degradation at high concentrations.

### Conclusions

A continuous flow-system for the on-line monitoring of parameters of interest in fish farms is proposed. The monitoring can be based on the direct measurement of sample features, or the measurements of the product of a derivatizing reaction. Both individual and in-series detectors are used, depending on the parameter to be monitored.

Although the arrangement has been applied to the determination of the most common parameters in the aquaculture field, they can be altered by changing the detector and calibration data (direct monitoring) or by changing the program of the switching valve and the calibration data for parameters which require a prior derivatizing reaction. Another type of detector can also be used in the latter case, depending on the type of chemical system chosen. So, the versatility of the approach allows its easy adaptation to different systems.

The fully automated manifold allows long periods of unattendance. In addition, an alarm system can be fitted for cases where the levels of the different analytes/parameters are out with the suitable range.

### Acknowledgement

The authors wish to thank the Comisión Interministerial de Ciencia y Tecnología (CICyT) for its financial support (under Grant No. MAR88-0112).

### References

1. Luque de Castro, M.D., *Talanta*, **38** (1989), 591–599.
2. Goto, M., *Trends Analytical Chemistry*, **2** (1983), 92–94.
3. Valcárel, M. and Luque de Castro, M.D., *Flow Injection Analysis: Principles and Applications* (Ellis Horwood, Chichester, 1987).
4. Ruzicka, J. and Hansen, E. H., *Flow Injection Analysis* (Wiley and Sons, New York, 1988).
5. Linares, P., Luque de Castro, M. D. and Valcárel, M., *Journal of Automatic Chemistry*, **14**, 173–175 (1992).
6. *CRC Handbook of Chemistry and Physics*, 42nd edn (Chemical Rubber Company, Cleveland, 1989).

